# Diabetes and risk of peripheral artery disease in patients undergoing first-time coronary angiography between 2000 and 2012 – a nationwide study

**DOI:** 10.1186/s12872-019-1213-1

**Published:** 2019-10-24

**Authors:** Sadaf Kamil, Thomas S. G. Sehested, Nicholas Carlson, Kim Houlind, Jens F. Lassen, Casper N. Bang, Helena Dominguez, Christian T. Pedersen, Gunnar H. Gislason

**Affiliations:** 1 Department of Cardiology, Copenhagen University Hospital Herlev-Gentofte, Herlev and Gentofte Hospital, Kildegaardsvej 28, 2900 Hellerup, Denmark; 2 Department of Cardiology, Bispebjerg-Frederiksberg University Hospital, Copenhagen, Denmark; 3 Department of Biomedical Sciences, University of Copenhagen, Copenhagen, Denmark; 4 Department of Nephrology, Copenhagen University Hospital Rigshospitalet, Copenhagen, Denmark; 5 The Danish Heart Foundation, Copenhagen, Denmark; 6 Department of Vascular Surgery, Kolding Hospital, Kolding, Denmark; 7 Department of Regional Health Research, University of Southern Denmark, Odense, Denmark; 8 Department of Cardiology, Odense University Hospital, Odense, Denmark; 9 Department of Cardiology, Copenhagen University Hospital Rigshospitalet, Copenhagen, Denmark; 10 Department of Cardiology, Nordsjaellands Hospital, Hilleroed, Denmark

**Keywords:** Type 2 diabetes mellitus, Peripheral artery disease, Coronary artery disease, Atherosclerosis

## Abstract

**Background:**

The risk of peripheral artery disease (PAD) in patients with diabetes mellitus (DM) and coronary artery disease (CAD) is an important and inadequately addressed issue. Our aim is to examine the impact of DM on risk of PAD in patients with different degrees of CAD characterized by coronary angiography (CAG).

**Methods:**

Using nationwide registers we identified all patients aged ≥18 years, undergoing first time CAG between 2000 and 2012. Patients were categorized into DM/Non-DM group, and further classified into categories according to the degree of CAD i.e., no-vessel disease, single-vessel disease, double-vessel disease, triple-vessel disease, and diffuse disease. Risk of PAD was estimated by 5-year cumulative-incidence and adjusted multivariable Cox-regression models.

**Results:**

We identified 116,491 patients undergoing first-time CAG. Among these, a total of 23.969 (20.58%) had DM. Cumulative-incidence of PAD among DM patients vs. non-DM were 8.8% vs. 4.9% for no-vessel disease, 8.2% vs. 4.8% for single-vessel disease, 10.2% vs. 6.0% for double-vessel disease, 13.0% vs. 8.4% for triple-vessel disease, and 6.8% vs. 6.1% for diffuse disease, respectively. For all patients with DM, the cox-regression analysis yielded significantly higher hazards of PAD compared with non-DM patients with HR 1.70 (no-vessel disease), 1.96 (single-vessel disease), 2.35 (double-vessel disease), 2.87 (triple-vessel disease), and 1.46 (diffuse disease), respectively (interaction-p 0.042).

**Conclusion:**

DM appears to be associated with increased risk of PAD in patients with and without established CAD, with increasing risk in more extensive CAD. This observation indicates awareness on PAD risk in patients with DM, especially among patients with advanced CAD.

## Background

Peripheral artery disease (PAD) is characterized as narrowing of the arteries in the upper and lower extremities arteries [1]. Atherosclerosis is recognized as the most direct and important cause of PAD, leading to acute or chronic limb ischemia [2]. PAD is known to be one of the major complications of diabetes mellitus (DM) [3–6]. The Framingham heart study indicates that 20% of symptomatic PAD patients presents DM [7]. However, as PAD is mostly asymptomatic, a higher risk of DM in this group of patients is assumed [8, 9].

Several large population-based studies have indicated that DM is associated with a two- to four-fold increase in development of PAD compared to non-diabetic patients, moreover studies have shown a three- to four-fold increased risk of mortality in patients with DM and PAD compared to healthy individuals [10–13]. Additionally, patients with concomitant PAD and DM experience reduced quality of life and an increase in long-term disability and functional impairment [14, 15]. Screening for PAD is therefore advocated in the international guidelines [16]. Comparably, the European Association for the Study of Diabetes recommends annual screening in all patients regardless of risk factors [17]. Of note, PAD is associated with substantial increased risk of fatal and non-fatal cardiovascular and cerebrovascular events, plausibly due to a greater degree of overall systemic atherosclerosis [18, 19]. Thus, an early detection and timely treatment of clinical risk factors may contribute in reducing the prevalence and severity of PAD in patients with DM and coronary artery disease (CAD).

Nevertheless, the association of PAD with respect to the severity of cardiovascular calcification remains untested, and the impact of DM on development of PAD in patients with CAD needs further study. With the present study, we aimed to investigate the association of cardiovascular calcification with PAD in patients with and without DM undergoing de novo coronary angiography (CAG) in a real-world nationwide setting.

## Methods

### Study design and data sources

All Danish citizens have a unique and permanent civil registration number that enables individual-level linkage across all Danish nationwide registers [20–24]. In the present study, patients were identified by using the Danish Heart Registry (DHR), a clinical database containing information on all patients referred for CAG within the Danish healthcare system [25]. The Danish National Patient Registry holds information on all in-patient and out-patient treatments [recorded as International Classification of Diseases (ICD) codes] nationwide since 1978 and was used to retrieve information on comorbidities, prior to the CAG procedure date [20]. The Danish Register of Medicinal Product Statistics (National Prescription Register) holds information on all prescribed medicine dispensed since 1995 according to The International Anatomical Therapeutic Chemical (ATC) classification system and was used to obtain data on concomitant pharmacotherapy. These Danish registers have previously been shown to be complete and accurate [20, 21]. The present study is conducted and reported in accordance with the recommendation of Strengthening the Reporting of Observational Studies in Epidemiology (STROBE) [20].

### Inclusion criteria and classification of patients

This present study included all Danish citizens aged ≥18 years, undergoing first-time CAG over a period of 12 years spanning January 1, 2000 to December 31, 2012. Patients with more than one CAG within the study period were only included at their first appearance only. Moreover, the study subjects were censored on death, migration, and at the end of study period (December 31, 2012). The study population was then segregated into patients with DM and patients without DM (non-DM) with a further subdivision into five categories of CAD i.e. no-vessel disease, single-vessel disease, double-vessel disease, triple-vessel disease, and diffuse disease. Obstructive coronary artery disease (i.e. single-, double- or triple-vessel disease) was defined as one or more epicardial coronary arteries with > 50% angiographic lumen narrowing, whereas no-vessel disease was defined as angiographic lumen narrowing (< 50%) in a single coronary vessel. On the similar lines, patients registered with diffuse (coronary artery) disease includes those with diffuse non-significant atherosclerosis in more than one coronary artery or with non-obstructive (< 50%) lesions in multiple coronary vessels. Of notice, the degree of vessel disease was to the discretion of the invasive cardiologist.

### Outcome

PAD was the outcome of interest and was defined as all arterial diseases except the atherosclerotic disease of coronary arteries, aorta, and intracranial arteries. ICD-10 diagnoses codes (DI170, DI73, DI74) were used to identify patients with PAD. Moreover, the diagnoses of PAD registered in Danish National Patient Registry are validated with an over-all positive predictive value of 100% (CI 92.9–100) [26]. Patients with a history of PAD (*n* = 7.342) at baseline were excluded from the study cohort before the study start.

### Pharmacotherapy and comorbidity

Baseline pharmacotherapy was defined by dispensed prescriptions 180 days prior to CAG. Comorbidities were established based on diagnostic codes recorded in the National Patient Registry, including atrial fibrillation, hypertension, vascular disease, renal disease, and thromboembolism. Hypertension was identified by either a hospital diagnosis for hypertension, or concurrent use of at least 2 of the following classes of antihypertensive agents within a 3-month period: α-adrenergic blockers, non-loop diuretics, vasodilators, β-blockers, calcium-channel blockers, and renin-angiotensin system inhibitors. DHR records information on DM status, body mass index (BMI), and smoking status on all patients undergoing CAG. To increase the sensitivity of the exposure group, we defined DM by use of glucose-lowering agents and a diagnosis of DM retrieved from DHR and National Prescription Register, respectively. The respective ICD, ATC, codes for all examined comorbidities and concomitant medications are presented in Table 1.

**Table 1:** Overview of ICD, and ATC codes for all examined comorbidities and concomitant medications

Pharmacological Treatments	ATC
Cholesterol-lowering drugs	C10A
Acetylsalicylic acid	B01AC06
Glucose-lowering agents	A10
Vitamin K antagonists	B01AA
Digoxin	C01AA
Platelet inhibitors	B01AC
Hypertension	
Α-adrenergic blockers	C02A, C02B, C02C, C02DA, C02L, C03A, C03B, C03D, C03E, C03X, C07C, C07D, C08G, C09BA, C09DA, C09XA52, C02DB, C02DD, C02DG, C07, C07F, C08, C09BB, C09.
Non-loop diuretics	
Vasodilators	
Β-blockers	
Calcium channel blockers	
Renin angiotension system inhibitors	
Comorbidity	ICD-10
Vascular disease	I21 to I22
Atrial fibrillation	I48
Thromboembolism	I26, I63, I64, I74, G458, G459
Hypertension	I10 to I15
Renal Disease	N03, N04, N17 to N19, R34, I12, I13

### Statistical analysis

Baseline characteristics were presented as medians, frequencies and percentages. χ^2^-test was used to test the difference between categorical variables and t-test or the Kruskal-Wallis test for differences between continuous variables. The level of statistical significance was set as *p* < 0.05. Risk of PAD in 5-years of follow-up, between the respective subgroups were presented as cumulative incidence curves with 95% confidence interval (CI) using the Aalen-Johansen method. Risk-time was constituted of time since CAG procedure date until an outcome of PAD occurred. Multivariable Cox proportional-hazard models adjusted for sex, age, smoking status, BMI, hypertension, and concomitant medication (beta-blockers, vitamin K antagonist, platelet inhibitors, acetylsalicylic acid, and cholesterol lowering drugs), were used to estimate hazard ratios (HR) and confidence intervals of PAD between subgroups of patients with and without DM. Patients with no-DM and no-vessel disease were used as references.

All statistical analyses were performed with SAS statistical software version 9.4 (SAS Institute Inc., Cary, North Carolina, USA), R statistics (R Core Team, 2016), and Stata software version 14 (Statacorp, College St., Texas, USA).

## Results

**Fig. 1:**
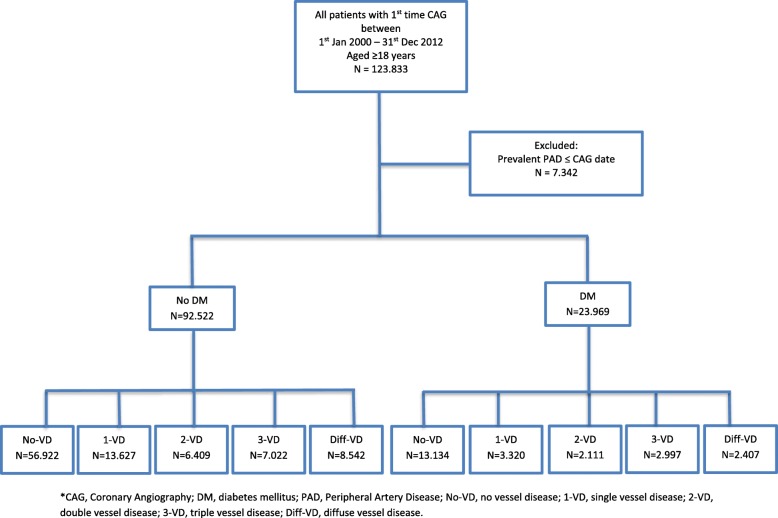
Flowchart of selection of study population

A total of 116,491 patients underwent first-time CAG between January 1, 2000 and December 31, 2012. Patients with previous known PAD by the start of the study (*n* = 7.342) were excluded. A total of 23,969 (20.58%) were identified with DM. Mean age was 64.31 [IQR 56–73 years], and 63.67% of the identified patients were male. A flowchart of the study population is illustrated in Fig. 1, and baseline characteristics stratified by the degree of CAD among patients with and without DM are presented in Table 2.

**Table 2:** Baseline characteristics of the study population

	Non-diabetes					Diabetes				
	No CAD	Single vessel disease	Double vessel disease	Triple vessel disease	Diffuse disease	No CAD	Single vessel disease	Double vessel disease	Triple vessel disease	Diffuse disease
N (%)	56.922 (61.52)	13.627 (14.73)	6.409 (6.93)	7.022 (7.59)	8.542 (9.23)	13.134 (54.80)	3.320 (13.85)	2.111 (8.81)	2.997 (12.50)	2.407 (10.04)
Sex, male (%)	33.361 (58.61)	9.499 (69.71)	4.777 (74.54)	5.594 (79.66)	4.860 (56.90)	8.376 (63.77)	2.298 (69.22)	1.574 (74.56)	2.299 (76.71)	1.528 (63.48)
Age, mean (IQR*)	63.17 (55–72)	63.51 (55–72)	66.95 (59–75)	69.57 (63–77)	66.13 (59–74)	63.84 (57–72)	63.93 (57–72)	65.91 (59–73)	67.29 (61–74)	64.96 (58–72)
Smoking (%)	14.042 (24.67)	4.983 (36.57)	2.064 (32.20)	1.923 (27.39)	2.100 (24.58)	3.089 (23.52)	1.030 (31.02)	624 (29.56)	787 (26.26)	559 (23.22)
B-blockers* (%)	22.256 (39.10)	3.112 (22.84)	1.931 (30.13)	2.516 (35.83)	2.723 (31.88)	6.609 (50.32)	1.040 (31.33)	795 (37.66)	1.244 (41.51)	916 (38.06)
Statins (%)	22.529 (39.58)	3.360 (24.66)	2.078 (32.42)	2.771 (39.46)	2.981 (34.90)	8.515 (64.83)	1.462 (44.04)	1.011 (47.89)	1.611 (53.75)	1.332 (55.34)
ACE-I* (%)	18.914 (33.23)	3.076 (22.57)	1.817 (28.35)	2.277 (32.43)	2.632 (30.81)	8.008 (60.97)	1.470 (44.28)	985 (46.66)	1.600 (53.39)	1.307 (54.30)
Spironolacton (%)	2.314 (4.07)	250 (1.83)	179 (2.79)	225 (3.20)	296 (3.47)	1.048 (7.98)	125 (3.77)	103 (4.88)	188 (6.27)	158 (6.56)
Loop diuretics (%)	7.595 (13.34)	885 (6.49)	605 (9.44)	903 (12.86)	1.055 (12.35)	3.430 (26.12)	508 (15.30)	385 (18.24)	693 (23.12)	554 (23.02)
ASA* (%)	25.578 (44.94)	3.630 (26.64)	2.234 (34.86)	3.026 (43.09)	3.132 (36.67)	7.880 (60.00)	1.283 (38.64)	991 (46.94)	1.589 (53.02)	1.124 (46.70)
VKA* (%)	4.711 (8.28)	563 (4.13)	332 (5.18)	360 (5.13)	717 (8.39)	1.393 (10.61)	187 (5.63)	131 (6.21)	179 (5.97)	252 (10.47)
Platlet inhibitors (%)	26.598 (46.73)	3.849 (28.25)	2.371 (36.99)	3.208 (45.68)	3.346 (39.17)	8.187 (62.33)	1.374 (41.39)	1.042 (49.36)	1.690 (56.39)	1.207 (50.15)
Digoxin (%)	2.248 (3.95)	311 (2.28)	199 (3.11)	266 (3.79)	468 (5.48)	924 (7.04)	137 (4.13)	114 (5.40)	181 (6.04)	199 (8.27)
Atrial Fibrillation (%)	5.007 (8.80)	791 (5.80)	425 (6.63)	518 (7.38)	1.041 (12.19)	1.543 (11.75)	258 (7.77)	189 (8.95)	244 (8.14)	3.621 (15.04)
IHD* (%)	4.812 (8.45)	1.134 (8.32)	859 (13.40)	1.234 (17.57)	780 (9.13)	1.789 (13.62)	401 (12.08)	398 (18.85)	702 (23.42)	281 (11.67)
Renal Disease (%)	897 (1.58)	132 (0.97)	91 (1.42)	123 (1.75)	144 (1.69)	383 (2.92)	74 (2.23)	60 (2.84)	82 (2.74)	81 (3.37)
Hypertension (%)	9.099 (15.99)	1.772 (13.00)	1.029 (16.06)	1.290 (18.37)	1.877 (21.97)	4.115 (31.33)	871 (26.23)	599 (28.38)	932 (31.10)	916 (38.06)
Thromboembolus(%)	4.128 (7.25)	893 (6.55)	511 (7.97)	763 (10.87)	829 (9.70)	1.308 (9.96)	326 (9.82)	228 (10.80)	409 (13.65)	273 (11.34)

Continuous variables are presented as means (with standard deviation values) and discrete variables as percentages (%). *B-blocker* Beta-blocker, *ACEI* Angiotensin converting enzyme inhibitors*, ASA* Acetylsalicylic acid, *VKA* Vitamin K antagonist

*Beta-Blockers *Angiotensin Converting Enzyme Inhibitors *Acetylsalicylic Acid *Vitamin K Antagonists *Ischemic Heart Disease *Interquartile range

Table 3 demonstrates number of patients (given in percentages) with DM and non-DM in categories of CAD showing a higher percentage of double, triple, and diffuse-vessel disease among patients with DM, compared to non-DM patients.

**Table 3:** Percentages of CAD in patients with and without DM

Degree of CAD	Non-DM	DM
No vessel disease	61.52%	54.80%
Single vessel disease	14.73%	13.85%
Double vessel disease	6.93%	8.81%
Triple vessel disease	7.59%	12.50%
Diffuse vessel disease	9.23%	10.04%

The primary endpoint i.e. PAD was diagnosed in 4.737 patients (4.07%) in a 5-year follow-up period with a higher percentage among patients with DM (6.15%) compared to non-DM patients (3.53%).

**Fig. 2:**
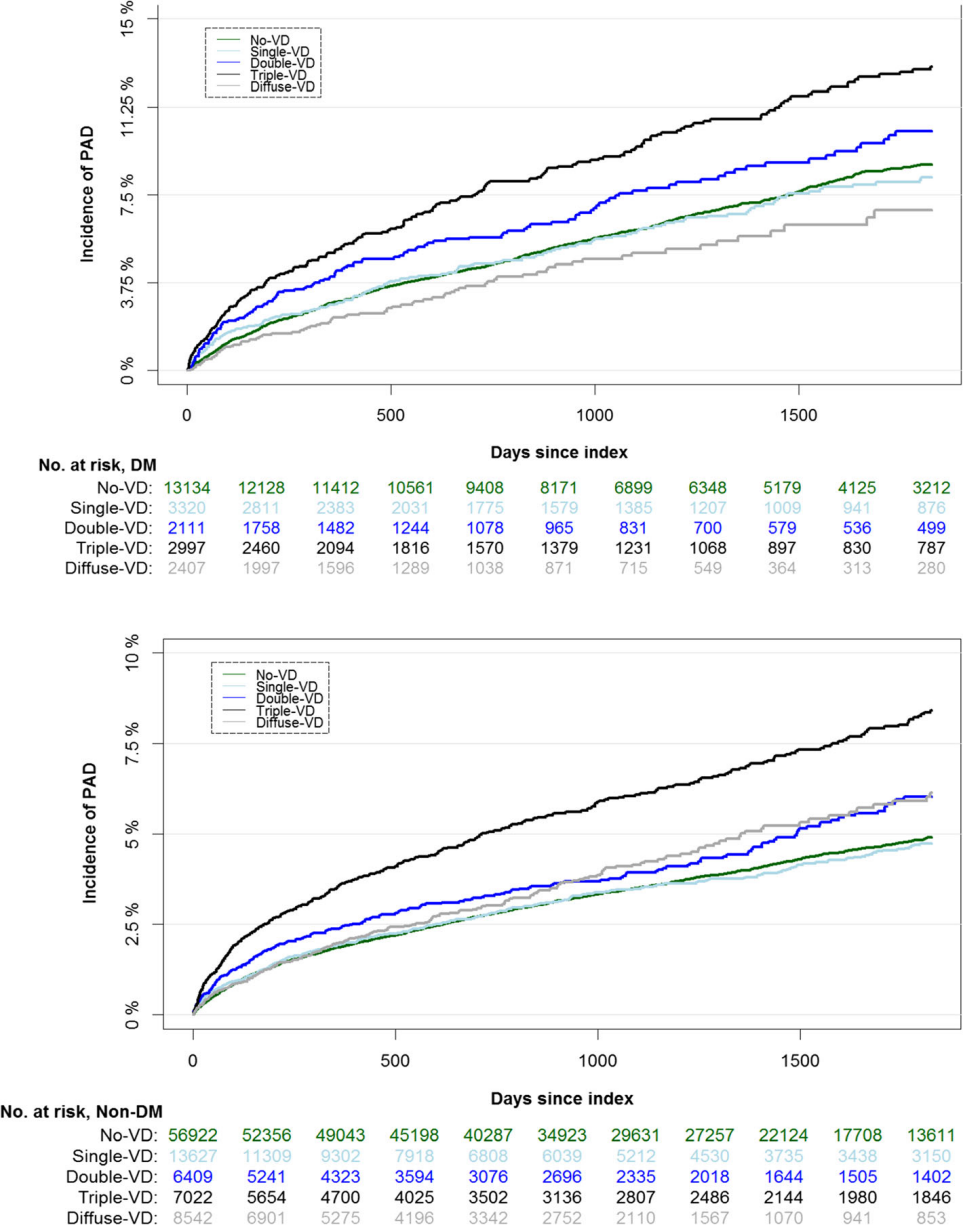
Cumulative Incidence Curve and patients at risk within 5 years from coronary angiography stratified by degree of CAD in patients with DM compared to non-DM patients. At risk table represents number of patients at risk of event at given time

The 5-year cumulative incidence of PAD among DM patients were 8.8, 8.2, 10.2, 13.0, and 6.8% in no-vessel disease, single-vessel disease, double-vessel disease, triple-vessel disease, and diffuse disease, respectively. Whereas, the corresponding cumulative incidence of PAD among patients with non-DM were 4.9, 4.8, 6.0, 8.4, and 6.1% in no-vessel disease, single-vessel disease, double-vessel disease, triple-vessel disease, diffuse disease, respectively (Fig. 2).

Correspondingly, the age- and sex-adjusted cox regression models yielded increased hazards of PAD among DM patients with HR 1.73 (1.59–1.87), 1.91 (1.63–2.24), 2.37 (1.99–2.83), 2.94 (2.57–3.37), and 1.59 (1.28–1.97) in no-vessel disease, single-vessel disease, double-vessel disease, triple-vessel disease, and diffuse disease, respectively. On the contrary, hazards of PAD in non-DM patients were comparably lower with HR 1.0, 1.12 (0.92–1.05), 1.30 (1.17–1.36), 1.77 (1.53–1.72), and 1.36 (0.92–1.06) in no-vessel disease, single-vessel disease, double-vessel disease, triple-vessel disease, diffuse disease, respectively. P for interaction between DM and degree of CAD, compared to non-DM was 0.042. Furthermore, the fully adjusted HRs remained significant, showing an increased risk of PAD in patients with DM that appears to increase with the severity of CAD (Table 4).

**Table 4:** Hazard ratios and 95% confidence intervals of PAD in patients with and without DM with respect to degree of CAD along with individual covariates

	Hazard Ratio	95% Confidence Interval	*P*-Value
Non-DM			
No-CAD (reference)	1.0	NA	NA
Single-vessel disease	1.15	1.03–1.29	0.013
Double-vessel disease	1.37	1.18–1.58	< 0.001
Triple-vessel disease	1.82	1.61–2.05	< 0.001
Diffuse-vessel disease	1.33	1.16–1.52	< 0.001
DM			
No-CAD	1.70	1.55–1.86	< 0.001
Single-vessel disease	1.96	1.67–2.31	< 0.001
Double-vessel disease	2.35	1.96–2.82	< 0.001
Triple-vessel disease	2.87	2.50–3.30	< 0.001
Diffuse-vessel disease	1.46	1.17–1.84	< 0.001

## Discussion

In a nationwide register-based cohort study on all patients undergoing CAG, DM was observed to be independently associated with increasing risk of PAD dependent on severity of CAD. Moreover, the over-all associated risk of PAD remained significantly increased in patients with DM compared to non-DM patients.

PAD is defined as atherosclerosis of the lower extremity arteries and it is associated with increased risk of lower extremity amputation and impaired life quality [27]. Studies have linked this group of patients with a higher risk of DM [8–10, 28]. DM-associated atherosclerosis affects all major vascular beds, including cardiovascular system and arteries of the lower extremity and thereby contributes to cardiovascular disease burden and mortality [29]. An estimated 15% of patients with DM are reported to have PAD within 10 years after the time they are diagnosed with DM, and the number increases to 45% after 20 years [19]. PAD in patients with DM causes long-term disability including ischemic ulcer or gangrene, which ultimately may result in limb amputations [30]. The American Diabetes Association consensus panel recommends screening for PAD in all DM patients above the age of 50, or if they have risk factors such as smoking, dyslipidemia, and hypertension [16].

Although some studies have addressed this subject, results are based on limited data [18, 31, 32] and larger population based studies are warranted. We, therefore, used Danish national registers to examine the risk of PAD in patients with DM compared to non-diabetics and its relationship to CAD in a nationwide setting. The results in the present study confirm previous observations of an augmented risk of developing PAD in patients with DM and CAD, compared to patients without DM [31, 32]. Furthermore, this risk increases by the severity of CAD in patients with and without DM. However, in our study patients with diffuse disease in DM patients appears to have a lower cumulative incidence of PAD compared to no-vessel disease (Fig. 2). As the cumulative incidences of an event of interest are calculated in the presence of a competing risk (e.g. death), this variance in diffuse disease group could be explained by the substantial number of patients that die during the follow-up without experiencing the study outcome. Moreover, the HRs of PAD in patients with DM who suffer diffuse disease compared to no-vessel disease are not significantly different. Also, compared to diffuse and obstructive coronary artery disease, the proportion of patients with no-vessel disease was found to be higher in patients with DM (54%) and non-DM (63%). The degree of vessel disease recorded in DHR was subject to the discretion of the invasive cardiologist, which could result in more patients with borderline angiographic lumen narrowing (i.e. slightly higher than 50%) that are most likely registered as no-vessel disease. Of notice, patients at risk of developing CAD (including patients with DM) are most likely to be referred at earlier course of their disease, which may also explain a higher proportion of diffuse, no-vessel or single-vessel disease. Indeed, this notion is supported by the results demonstrated by Danish Health Authority (Sundhedsstyrelsen) and Danish Heart Foundation (Hjerteforeningen) indicating an increasing trend in number of coronary angioplasties per million Danish inhabitants [33].

DM is characterized by hyperglycemia, insulin resistance, and dyslipidemia that contribute to development and progression of PAD, through pathophysiological mechanisms (e.g. vascular inflammation, endothelial dysfunction, hemostasis dysregulation) similar to those in CAD [12, 34]. Vascular inflammation is a risk marker of atherothrombosis, and studies have shown that CAD patients with an additional diagnosis of PAD have higher circulating levels of inflammatory biomarkers [9, 35]. In view of that, the vascular inflammation caused by DM may play a contributing role in development of PAD by accelerating the disease process in patients with known CAD [12]. In addition, choice of glucose-lowering agents may have an important impact on the development of atherosclerotic plaques [36]. Moreover, risk of developing PAD in patients with concurrent DM and advance CAD also depend on interplay between factors such as comorbidities, smoking, obesity, and reduced physical activity [11]. These factors foster development of atherothrombosis and they are often present before the diagnosis of DM and worsen in line with the duration of DM [37]. Moreover, due to the distal territory of vessel involvement and its association with peripheral neuropathy, PAD is more commonly asymptomatic in patients with DM and may present later at a more advanced stage [38]. Also, the lower resistance to infection of this group dramatically increases their risk of amputation compared to non-diabetic patients with PAD [30]. Thus, an early detection and timely treatment of clinical risk factors may contribute in reducing the prevalence and severity of PAD in patients with DM and CAD.

Taken together, results from the present study add to the existing evidence that patients with concurrent DM and CAD may be at increased risk of developing PAD and this risk appears to worsen in advance CAD. Clinicians should be aware and may want to consider screening and treatment at early stage to elicit the adverse outcomes, specifically in high risk population of patients with DM and CAD. Nevertheless, prospective studies aimed to explore effects of such outcomes are required.

### Strengths and limitations

Large number of unselected patients in a real-world nationwide setting, completeness of follow-up, and use of validated measures of exposure and outcome are among the strengths of the present study. Furthermore, healthcare in Denmark is equally accessible to all Danish citizens minimizing confounding by variables associated with social class. It is our opinion that this large-scale nationwide study provides a reliable image of the CAG population under investigation.

There are also several limitations in the study that must be acknowledged. The observational nature of the study only allows to establish association and does not represent cause-and-effect relationships. Moreover, the subpopulation was identified by using diagnosis from registers along with data on claimed prescriptions. Although the registration of data in Danish registers is thought to be accurate, there might be some limitations regarding the PAD diagnoses; especially as PAD is underreported due to the often asymptomatic nature of the disease. Also, development of neuropathy in patients with DM often suppresses PAD symptoms leading to late diagnosis of PAD. In opposite, patients with DM are followed closely with regularly health checkups that might lead to earlier detection of PAD. The registries used in this study do not include information on glycosylated hemoglobin levels and patients with DM managed with diet alone or developing DM during the follow up period were not identified. To address this issue, we defined DM by use of glucose lowering agents and the recorded diagnoses of DM in DHR and National Prescription Register, respectively. Nevertheless, the results are only valid for patients with DM requiring pharmacotherapy at the time of CAG. Moreover, DHR lacked information on various clinical parameters including thorough data on the luminal diameter and precise extent of coronary artery stenosis (in percentage).

## Conclusion

In this observational nationwide study of patients undergoing CAG, DM was associated with increased risk of PAD in patients with and without established CAD. Additionally, we observed increasing risk of PAD with more extensive CAD. Further studies are warranted to support these findings.

## Data Availability

The datasets generated and/or analysed during the current study are not publicly available but are accessible on reasonable request and through permission from DST (Danmark statistik).

## Abbreviations

BMI: Body mass index
CAD: Coronary artery disease
CAG: Coronary angiography
DM: Type 2 diabetes mellitus
HR: Hazard ratio
IR: Incidence rate
PAD: Peripheral artery disease
